# Enhanced Oral Bioavailability of Efavirenz by Solid Lipid Nanoparticles: *In Vitro* Drug Release and Pharmacokinetics Studies

**DOI:** 10.1155/2014/363404

**Published:** 2014-05-21

**Authors:** Praveen Kumar Gaur, Shikha Mishra, Meenakshi Bajpai, Anushika Mishra

**Affiliations:** ^1^Department of Pharmaceutics, I.T.S. Paramedical College (Pharmacy), Muradnagar, Ghaziabad 201206, India; ^2^Department of Pharmacognosy & Phytochemistry, Jamia Hamdard, New Delhi 110062, India

## Abstract

Solid lipid nanoparticle is an efficient lipid based drug delivery system which can enhance the bioavailability of poorly water soluble drugs. Efavirenz is a highly lipophilic drug from nonnucleoside inhibitor category for treatment of HIV. Present work illustrates development of an SLN formulation for Efavirenz with increased bioavailability. At first, suitable lipid component and surfactant were chosen. SLNs were prepared and analyzed for physical parameters, stability, and pharmacokinetic profile. Efavirenz loaded SLNs were formulated using Glyceryl monostearate as main lipid and Tween 80 as surfactant. ESLN-3 has shown mean particle size of 124.5 ± 3.2 nm with a PDI value of 0.234, negative zeta potential, and 86% drug entrapment. *In vitro* drug release study has shown 60.6–98.22% drug release in 24 h by various SLN formulations. Optimized SLNs have shown good stability at 40°C ± 2°C and 75 ± 5% relative humidity (RH) for 180 days. ESLN-3 exhibited 5.32-fold increase in peak plasma concentration (*C*_max⁡_) and 10.98-fold increase in AUC in comparison to Efavirenz suspension (ES).

## 1. Introduction

Nanotechnology is the technology achieved on nanoscale having the application in the real world. It encompasses the production and application of physical, chemical, and biological systems at submicron dimensions as well as the integration of the resulting nanostructures into larger systems [1–4].

Solid lipid nanoparticles (SLNs) were developed at the beginning of the 1990s as submicron colloidal carriers (50–1000 nm) made up of lipids (Compritol 888 ATO, Dynasan 112, beeswax, carnauba wax, emulsifying wax, cetyl alcohol, cholesterol butyrate, and cholesterol) and stabilized by surfactant [5]. SLNs are at the forefront of the rapidly developing field of nanotechnology due to possibility for site specific drug delivery, controlled release, increased bioavailability, reduced side effects, smaller dosage form, dosage form stability, and reduction in fed/fasted variability [6, 7]. The SLN's ability to incorporate hydrophilic/hydrophobic drugs imparts unique diversity. Hence controlled drug delivery, enhancement of bioavailability of entrapped drugs via modification of dissolution rate and/or improvement of tissue distribution, and targeting of drugs by using SLNs have been reported in various application routes like parenteral (intravenously, intramuscularly, or subcutaneously), oral, rectal, ophthalmic, and topical (cosmetics and dermatological) preparations [8–11].

Efavirenz is a nonnucleoside reverse transcriptase inhibitor and is used as first line antiretroviral drug in the high activity antiretroviral therapy (HAART) for the human immunodeficiency virus (HIV) infections. It belongs to BCS class II category with high lipophilicity (log *P* = 5.4), poor aqueous solubility (4 *μ*g/mL), low intrinsic dissolution rate (0.037 mg/cm^2^/min), limited oral bioavailability (40–50%), and high intersubject variability [12, 13]. It is currently available as tablet (600 mg) or capsules (50 mg or 200 mg) (Sustiva) [13]. Solubility enhancement can improve the bioavailability as dissolution is the rate limiting step in its absorption so the aim of this work was to develop SLN to improve the solubility and bioavailability of Efavirenz after oral administration.

## 2. Materials and Methods

### 2.1. Materials

Efavirenz was the gift sample from Jubilant Clinsys Noida, India, whereas Glyceryl monostearate (1-stearoyl-rac-glycerol), stearic acid (octadecanoic acid), and Tween 80 (polysorbate 80) along with all the other chemicals were of analytical grade and were purchased from Sigma-Aldrich (New Delhi, India). Compritol ATO 888 and Precirol were the gift sample from Asoj Soft Caps, Baroda, India. Commercial formulation was EFCURE oral solution (Emcure Pharmaceuticals Ltd.) containing Efavirenz (30 mg/60 mL).

### 2.2. Methods

#### 2.2.1. Preformulation Studies

*Estimation of Drug Solubility*. Suitable quantities of drug were added in the solvent and a saturated solution was obtained. Then the resultant solution was filtered and assayed by HPLC.

*Determination of Partition Coefficient*. The partition coefficient was determined by shake flask method. Suitable quantity of drug was added in n-octanol and a saturated solution was obtained. Then equal quantity of water was added and the mixture was shaken vigorously. This mixture was allowed to stand for 24 hours. After that, the two phases were separated and drug content in each phase was determined by HPLC.

## 3. Formulation Development

### 3.1. Excipient Selection

Lipid and surfactant are critical components of solid lipid nanoparticles. So, suitable lipid and surfactant components were selected. Initially, the solubility of Efavirenz was determined in various lipids (Table 1). The lipid was melted and suitable quantity of drug was added to it. The addition of drug was continued till a clear pale solution was obtained. Then, this drug lipid mixture was dissolved in methanol and filtered through 0.22 *μ*m filter. The drug content was then analyzed by HPLC.

Further, the content of surfactant was optimized by making SLNs with varying surfactant concentration (0.5%–1.25%) and analyzing for various physicochemical parameters.

*Preparation of SLN*. 150 mg of GMS was dissolved in 10 mL organic solvent (1 : 1 chloroform and methanol) and 50 mg of drug was dispersed in this lipid solution. Organic solvent was removed by using rotary evaporator. Drug embedded lipid layer was melted by heating at 5°C above melting point of the lipid [3, 7, 14]. Simultaneously, an aqueous phase was prepared by dissolving Tween 80 in Milli-Q water and heated to same temperature. Hot aqueous phase was added to the lipid phase with continuous stirring at 3000 rpm for 30 min. The mixture was homogenized for 4 hours. After that, SLNs were filtered and dried (Table 2).

### 3.2. Drug-Excipients Interaction Study

1 mg of the sample and 300 mg of KBr were taken in a mortar and triturated. A small amount of triturated sample was taken into a pellet maker and was compressed at 1000 kg/cm^2^. The pallet was kept onto the sample holder and scanned from 4000 cm^−1^ to 400 cm^−1^. Initially, IR spectra of drug were taken and then 1 : 1 ratios of drug + lipid, drug + surfactant, and drug + lipid + surfactant were evaluated.

### 3.3. Efavirenz Assay

The drug content was assessed using HPLC instrument consisting of a Shimadzu LC-10AT VP pump, a SIL-10AF autoinjector, an SPD-10A UV-VIS detector, and an SCL-10A VP system controller equipped with Shim-pack VP-ODS column (Shimadzu, Japan). The column dimensions were 4.6 mm i.d. and 150 mm bed length with 5 *μ*m sized adsorbent. The sample was diluted in methanol and 20 *μ*L was injected into the column [6, 15, 16]. The column was eluted isocratically with acetonitrile and pH 7.4 ammonium acetate buffer (50 : 50, v/v) at 1.0 mL/min. The detection wavelength was set at 246 nm.

## 4. Characterization

### 4.1. Shape and Surface Morphology

Shape and surface morphology of the solid lipid nanoparticles were visualized by scanning electron microscopy (SEM). The samples for SEM were prepared by lightly sprinkling nanoparticles on a double adhesive carbon tape, which was stuck to an aluminum stub. The stubs were then coated with gold to a thickness of 200 to 500 Å under an argon atmosphere using gold sputter module in a high vacuum evaporator. The samples were then randomly scanned and photomicrographs were taken at different magnifications.

### 4.2. Particle Size and Size Distribution

Photon correlation spectroscopy (PCS) is the most powerful technique for the measurement of particle size. 1 mL of SLN suspension was diluted to 10 mL with distilled water and average particle size and polydispersity index were measured by PCS.

### 4.3. Zeta Potential Measurements

The surface charge of solid lipid nanoparticles is denoted as zeta potential. It was determined by the electrophoretic mobility of solid lipid nanoparticles in U type tube at 25°C, using Zetasizer (Malvern, UK).

### 4.4. Drug Entrapment Efficiency

A fixed quantity of SLNs suspension (10 mL) was centrifuged at 18000 rpm for 30 min at 20°C (SARTORIOUS F-18 K). Then, the lipid portion was isolated and the absorbance of the drug in the supernatant was determined by HPLC at 246 nm. The drug entrapment of solid lipid nanoparticle was calculated by the following equation [7, 17, 18]:

 % drug entrapment efficiency = analyzed weight of drug in SLN × 100/theoretical weight of drug loaded in SLN.

### 4.5. *In Vitro* Drug Release

The drug release was performed by dialysis bag method. The dialysis bag retains nanoparticles and allows the free drug into the dissolution media with a cut-off of 14 KDa. The bag was soaked in double-distilled water for 12 h before use. 2 mL SLN dispersion was poured into the bag with the two ends fixed by clamps. The bags were placed in a conical flask filled with 50 mL phosphate buffer (pH 7.4), The conical flasks were placed into a thermostatic shaker at 37°C at a rate of 140 movements per min. Suitable aliquots were withdrawn at selected time intervals and volume was replaced with fresh medium [19, 20]. Aliquots were filtered through 0.22 *μ*m membrane filter and assayed by HPLC method.

### 4.6. Stability Studies

Stability testing provides indication about variation in quality of an active substance or pharmaceutical product under the influence of environmental conditions.

Stability was analyzed for selected formulations by keeping them at 40°C ± 2°C and 75 ± 5% relative humidity (RH) in stability chamber (Hicon, Delhi, India) for 180 days and then analyzing physical parameters and* in vitro* drug release [3, 4, 6, 21, 22].

### 4.7. Pharmacokinetic Studies

Pharmacokinetics study was performed on 8–10 weeks old albino rats. The experimental procedure was reviewed and approved by institutional animal ethics committee. Eighteen albino rats (average weight: 300 g) were divided in three groups and kept under standard laboratory conditions (temperature: 25 ± 2°C; relative humidity: 55 ± 5%), in polypropylene cages with free access to standard laboratory diet (Lipton feed, Mumbai, India) and water* ad libitum*. Animals were administered their respective treatments (Table 6) and blood samples (0.2 mL) were collected at predetermined time intervals till 24 hours [11, 23]. Plasma was separated by centrifuging the collected sample at 5000 rpm for 20 min and stored at −21°C until drug estimation using HPLC.

 Group I = ES (~10 mg).
 Group II = commercial formulation (~10 mg).
 Group III = ESLN-3 (~10 mg).

### 4.8. Data Analysis

The experiments were performed in triplicate and experiments involving animals were analyzed using data from six experiments. Statistical analyses were performed using the GraphPad Prism version 4 software by means of analysis of variance (ANOVA) or the paired* t*-test, where appropriate, and statistical significance was set at *P* < 0.05.

Plasma concentration (*μ*g) versus time (hrs) profiles was prepared and peak plasma concentration (*C*_max⁡_) and time of its occurrence (*t*_max⁡_) were read directly from the respective profiles. Area under concentration time curve (AUC_0→*t*_) was calculated according to linear trapezoidal method using GraphPad Prism version 4.

## 5. Results

### 5.1. Excipient Selection and Optimization

The foremost criteria for selection of materials for formulation development are pharmaceutical acceptability, nonirritant and nonsensitizing nature, and their generally regarded as safe (GRAS) categorization. Further requirements for SLNs formulation are higher solubility of the drug in the nonaqueous phase to maintain the drug in solubilized form. GMS was taken as main lipid phase for preparing the SLN as the solubility of Efavirenz was found to be highest in Glyceryl monostearate as compared to other lipids. Optimum combination of low and high hydrophilic lipophilic balance (HLB) surfactants is required for the formation of a stable formulation. In this study, Tween 80 (HLB 15) was selected as the surfactant. It has been reported in the literature that use of Tween 80 yields finer sized SLN.

After selection of components, the composition of formulation was optimized by selecting appropriate proportion of surfactant and lipid in formulation. The formulation was prepared using different amount of surfactant and particle size and zeta potential of respective formulations were evaluated (Tables 1 and 2). Drug lipid ratio is a vital parameter because higher lipid phase increases the entrapment efficiency; however, an upper limit is important to maintain the size of nanoparticle in a reasonable range.

### 5.2. Drug Excipients Interaction Study

**Figure 1:**
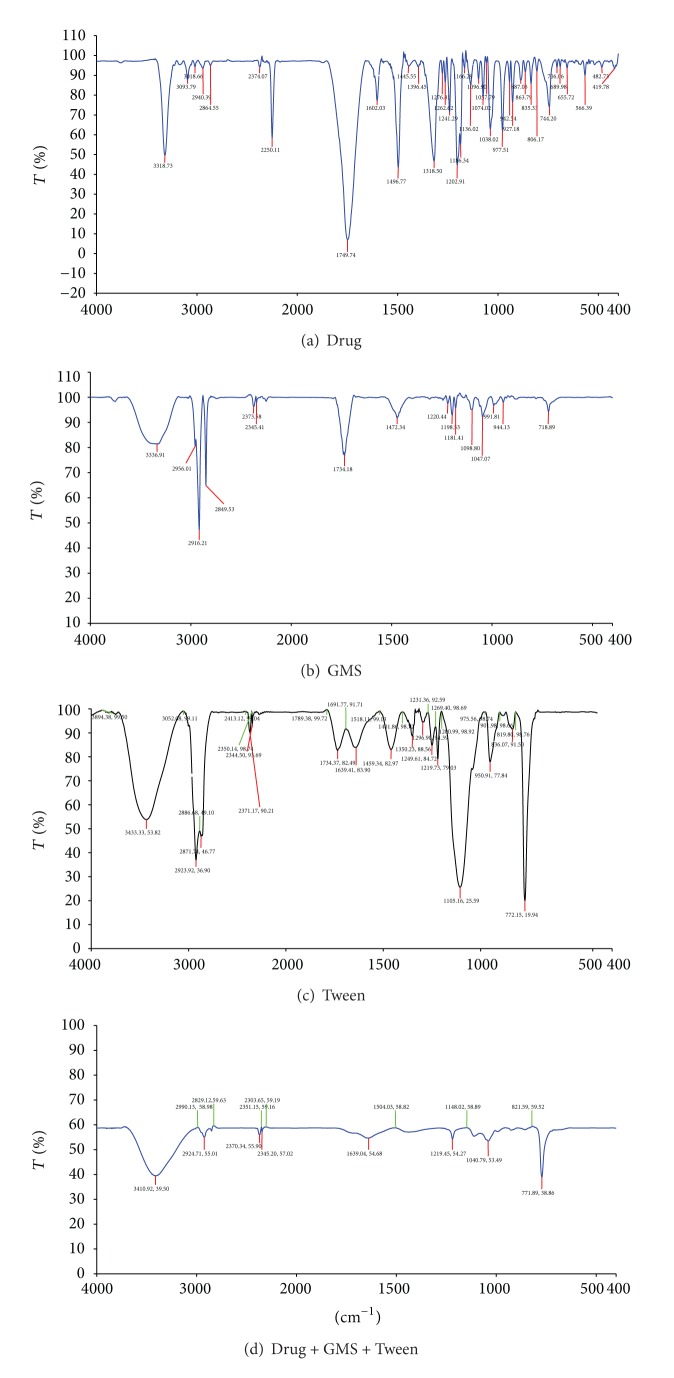
Drug excipients interaction studies by infrared spectra.

Drug excipients interaction was studied using IR spectroscopy. The spectra for physical admixture show shifts in IR peaks but when entrapment efficiency studies were performed, the authors got the drug solution for which HPLC was done for determining drug content. A separate IR evaluation was also run for this sample and IR spectra were comparable with pure drug. Based on above observation, it was inferred that the complex formed by drug and excipients is transient and will liberate active drug when released in physiological system (Table 3 and Figure 1).

### 5.3. Characterization of Formulations

The mean particle size of SLN formulations ranges from 124.5 ± 3.2 nm–362 ± 1.2 nm. The particle size of the formulation ESLN-3 was appreciably lower (124.5 ± 3.2 nm) compared to other formulations. This result is in accordance with the report that the addition of surfactant to solid lipid nanoparticle systems causes the interfacial film to condense and stabilize. All the formulations had particles in the nanorange which is well evident from the values of polydispersity. Polydispersity is basically the ratio of standard deviation to the mean particle size. All formulations had low values of polydispersity (0.234–0.455) indicating the uniformity of particle size (Table 4).

**Figure 2:**
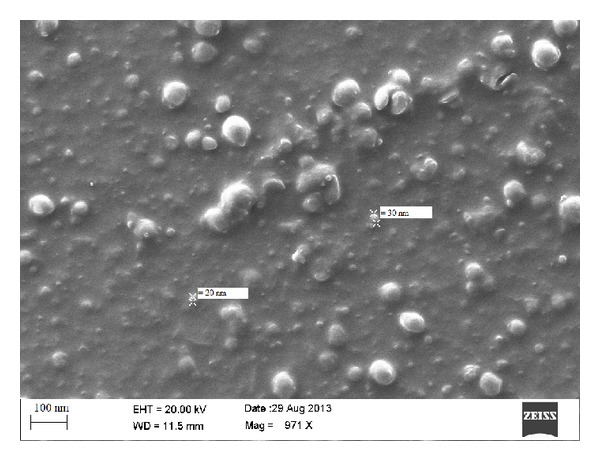
SEM images for ESLN-3.

**Figure 3:**
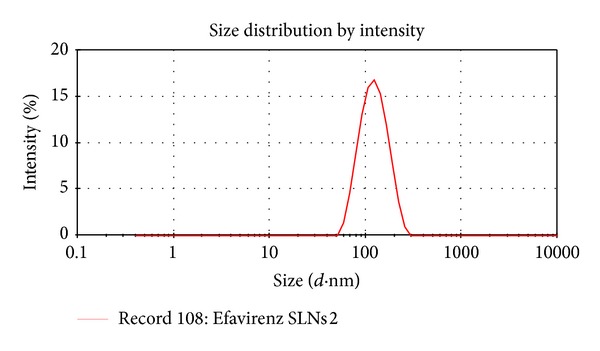
Size distribution (ESLN-3).

**Figure 4:**
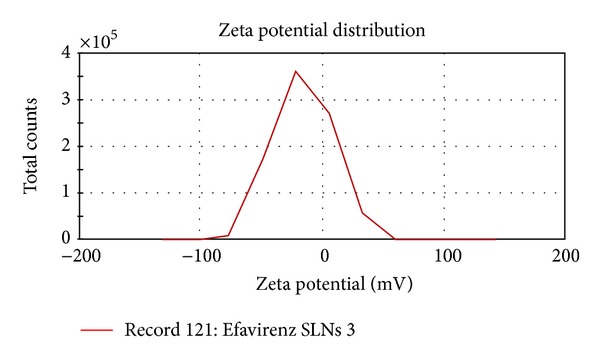
Zeta potential distribution (ESLN-3).

The zeta potential indicates the degree of charge present on suspended particles in dispersion. A suitably high value of zeta potential (positive or negative) confers stability because particles resist aggregation. All the studied formulations have shown the value of zeta potential between −15.9 to −22.1 (Figures 2, 3, and 4).

Drug entrapment of all the formulations was found to be 70.2 ± 1.2–86 ± 2.2% (Table 4). The image of SLN (ESLN3) was observed using SEM as shown in Figure 2. It shows smooth texture of surface morphology along with uniform shape and size.

### 5.4. *In Vitro* Drug Release Studies

**Figure 5:**
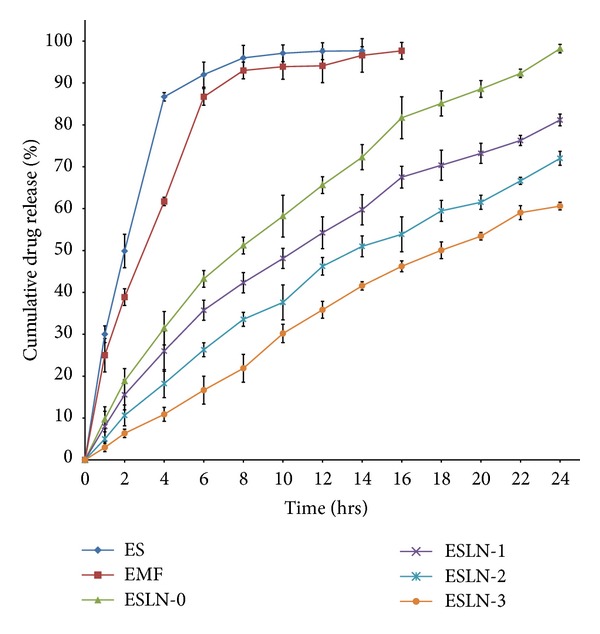
*In vitro* drug release from SLN, ES, and EMF.

*In vitro *drug release study of SLN (ESLN-3) along with EMF and ES was performed in phosphate buffer (pH 7.4). ESLN formulations have shown 60.6–98.22% drug release in a time period of 24 hrs whereas EMF and ES have shown 86.705% and 61.705% drug release in initial 4 hours, respectively (Figure 5).

### 5.5. Stability Studies

All the SLN formulations (ESLN-3) were evaluated for stability for 180 days. Most notifiable changes were observed in ESLN-0 whereas ESLN-3 showed almost negligible alteration in 180 days. So, it can be said that ESLN-3 was most stable formulation (Table 5).

### 5.6. Pharmacokinetic Studies

Pharmacokinetic parameters for the developed formulations were evaluated in rats after a single oral administration of ESLN-3, EMF, and ES by constructing respective plasma concentration time profiles using one compartment model analysis (Table 6).

**Figure 6:**
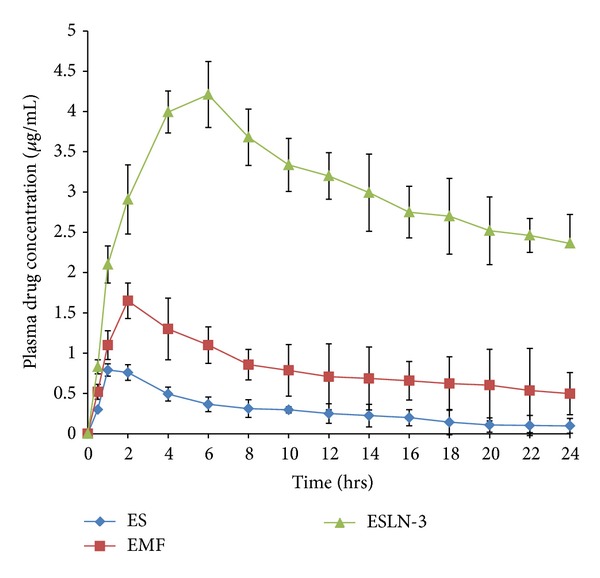
Plasma drug concentration time profiles.

ES has shown peak plasma concentration and AUC_0→24_ at 0.791 *μ*g/mL and 6.958 *μ*g/mL·h, respectively, whereas ESLN-3 has shown significant (*P* < 0.05) enhancement in the *C*_max⁡_ (4.21 *μ*g/mL) and AUC_0→24_ (76.4 *μ*g/mL·h). ESLN-3 exhibited 5.32-fold increase in peak plasma concentration (*C*_max⁡_) and 10.98-fold increase in AUC_0→24_ in comparison to ES. EMF has been taken for demonstrative purposes only (Figure 6).

## 6. Discussion

Efavirenz is a first line therapy in AIDS infection and it is available in the form of tablets and capsules in the market; however, owing to its limited aqueous solubility it has variable bioavailability [12, 13, 24]. In this work, a suitable lipid based formulation was developed to increase its bioavailability. For the preformulation part, the solubility of Efavirenz was determined in various aqueous and nonaqueous solvents and its partition coefficient was determined. The drug was freely soluble in ethanol, soluble in acetone, soluble in chloroform, soluble in methanol, and slightly soluble in distilled water. The partition coefficient of Efavirenz was 5.3, indicating the lipophilic nature of the drug. So, solid lipid nanoparticle formulation was selected due to its lipophilic nature because it shows better entrapment and less drug leakage.

Glyceryl monostearate and Tween 80 were selected as main lipid component and surfactant, respectively. ELSN-3 showed smallest particle size and it contained highest surfactant content. It has been reported that Tween 80 promotes formation of smaller sized nanoparticles. It was observed that increased content of surfactant promotes formation of smaller nanoparticles. All the SLN formulations showed negative zeta potential values which indicate the stable nature of nanoparticles owing to electrostatic repulsion. As the surfactant content increased, entrapment efficiency increases which could be due to formation of stabilized nanoparticles.

As evident from the* in vitro* drug release data, all the SLN formulations have shown controlled drug release. ES and EMF both have shown burst release. Since the drug is lipophilic in nature it diffuses through lipid core showing the sustained release. However the order of drug release is inversely proportional to the surfactant content which again highlights the stabilization role of surfactant. Since the higher surfactant content stabilizes the SLN, it also restricts the amount of drug released outside SLN.

The developed formulations have shown variable stability as the increased temperature affects the SLN more drastically. The most stable formulation was ESLN-3 which has shown optimum physicochemical parameters. The main source of instability in SLN is leaching of drug from SLN due to the movement of lipidic chains. However it has been established that incorporation of surfactant increases the stability of SLN [25] which in turn means less leaching of the drug from SLN. The amount of drug leached during storage can be interpreted in terms of loss in entrapment efficiency. The most stable formulation has shown the loss of 2.4% drug from the entrapped drug content. So it can be inferred that once an optimum mix of components is formulated, they complement each other and make for a stable formulation. Pharmacokinetic study has shown more clearly the controlled nature of SLN formulations as the selected SLN formulation achieves the highest plasma concentration at around 6 hours. Moreover, the peak plasma concentration was also increased by almost 5 times. This result can be explained on the basis of* in vitro* drug release. ES and EMF release almost 40% drug content in initial 2 hours so a major portion of dose goes in waste also reflecting in low values of AUC. The *C*_max⁡_ values for ES and EMF were low despite higher drug release which shows the poor nature of absorption from these formulations.

## 7. Conclusion

Present study shows the applicability of lipid based formulation in increasing the absorption of lipophilic drugs. GMS was used in the study because it has shown highest solubility for the drug. However the presence of surfactant is critical for formulating stable SLN formulation. In the present study, stable SLNs were formulated using appropriate proportions of GMS and Tween 80. The formulation ESLN-3 has shown optimally stable physicochemical parameters with higher values of *C*_max⁡_ and AUC.

## Figures and Tables

**Table 1:** Solubility of drug in different lipids.

Lipids	Solubility (g/g)
Compritol ATO 888	0.2374 ± 0.12
Glyceryl monostearate	0.4699 ± 0.031
Precirol	0.3760 ± 0.84
Stearic acid	0.3990 ± 0.19

All data expressed as mean ± S.D.; *n* = 3; *P* ≤ 0.05.

**Table 2:** Formulations composition.

Formulation code	Type of formulation	Drug : lipid	Surfactant (%)
ES	Suspension (1% drug)		
ESLN-0	SLN	1 : 3	0.5
ESLN-1	SLN	1 : 3	0.75
ESLN-2	SLN	1 : 3	1.0
ESLN-3	SLN	1 : 3	1.25

**Table 3:** Drug and excipients compatibility study by IR.

Components	Functional groups and wave number (cm^−1^)			
Drug	3318	2940.39	1749.74	1096, 1057, 1074
	(NH str)	(CH str)	(C=O str)	(C–O–C str)

Lipid (GMS)	3336.9	2916.21	1734.18	1098, 1047
	(OH str)	(CH str)	(C=O str)	(C–O–C str)

Surfactant	3433.33, 3453	2923.92, 2936.90	1734.37, 1782	1105.15, 1125.59
	(OH str)	(CH str)	(C=O str)	(C–O–C str)

Drug + lipid	3378.43	2933.59	1736.17	
	(OH str)	(CH str)	(C=O str)	

Drug + surfactant	3442.78	2923.50, 2926.37	1740.37, 1761.11	1103.68, 1119.59
	(OH str)	(CH str)	(C=O str)	(C–O–C str)

Drug + lipid + surfactant	3410.98, 3439.50	2924.71, 2955.01	1639.54, 1604.68	1040.79, 1053.59
	(OH str)	(CH str)	(C=O str)	(C–O–C str)

**Table 4:** Physical characterization.

Formulation code	PDI	Particle size	Zeta potential	EE (%)
ESLN-0	0.455	362 ± 2.1	−22.1	46.28 ± 1.05
ESLN-1	0.334	267 ± 2.2	−19.3	70.2 ± 0.98
ESLN-2	0.315	213.4 ± 2.4	−17.7	76.1 ± 0.7
ESLN-3	0.234	124.5 ± 3.2	−15.9	86 ± 1.03

All data expressed as mean ± S.D.; *n* = 3; *P* ≤ 0.05.

**Table 5:** Physical characterization of SLN after stability studies.

Formulation code	Days	Characterization parameters							
		Size (nm)		PDI		*ζ* potential (mV)		EE (%)	
		4°C	25°C	4°C	25°C	4°C	25°C	4°C	25°C
ESLN-0	0th	362 ± 2.1	362 ± 2.1	0.455	0.455	−22.1	−22.1	46.28 ± 1.5	46.28 ± 1.05
	30th	390 ± 1.4	398 ± 2.4	0.456	0.482	−21.9	−21.1	46.21 ± 1.3	42.87 ± 2.6
	90th	406 ± 1.7	428 ± 2.7	0.479	0.502	−21.7	−20.9	45.59 ± 1.27	38.92 ± 1.3
	180th	449 ± 1.1	534 ± 1.9	0.491	0.563	−21.5	−20.6	43.13 ± 1.9	34.28 ± 1.8

ESLN-1	0th	267 ± 2.2	267 ± 2.2	0.334	0.334	−19.3	−19.3	70.2 ± 0.98	70.2 ± 0.98
	30th	301 ± 1.6	316 ± 2.2	0.334	0.349	−19.1	−19.1	69.6 ± 0.4	68.8 ± 1.5
	90th	342 ± 2.1	372 ± 2.2	0.352	0.389	−19.1	−18.7	69.2 ± 1.3	67.3 ± 1.2
	180th	347 ± 1.3	427 ± 2.2	0.394	0.421	−19.1	−18.4	67.1 ± 1.4	65.6 ± 0.92

ESLN-2	0th	213.4 ± 2.4	213.4 ± 2.4	0.315	0.315	−17.7	−17.7	76.1 ± 0.7	76.1 ± 0.7
	30th	221 ± 1.1	234 ± 0.97	0.316	0.318	−17.7	−17.2	75.7 ± 0.91	74.5 ± 1.2
	90th	236 ± 1.6	278 ± 1.2	0.317	0.327	−17.2	−17.1	75.2 ± 1.4	73.3 ± 0.9
	180th	278 ± 2.2	302 ± 1.3	0.319	0.334	−17.1	−17.1	73.6 ± 1.2	72.6 ± 2.1

ESLN-3	0th	124.5 ± 3.2	124.5 ± 3.2	0.234	0.234	−15.9	−15.9	86 ± 1.2	86 ± 1.03
	30th	124.5 ± 1.1	124.8 ± 2.8	0.234	0.235	−15.7	−15.7	85.7 ± 1.1	85.3 ± 2.1
	90th	124.7 ± 2.3	124.9 ± 1.4	0.235	0.235	−15.7	−15.7	85.5 ± 1.5	84.3 ± 1.32
	180th	124.8 ± 1.4	125.2 ± 1.6	0.235	0.236	−15.5	−15.4	85.1 ± 1.3	83.6 ± 1.7

All data expressed as mean ± S.D.; *n* = 3; *P* ≤ 0.05.

**Table 6:** Pharmacokinetic studies of selected formulations.

Formulation	*C*_max⁡_	*T*_max⁡_	AUC	AUMC	*K*_el_	*T*_1/2_	MRT	RB
	(*μ*g/mL)	(hrs)	(*μ*g·hr/mL)	(*μ*g·hr^2^/mL)	(h^−1^)	(hr)	(hr)	(%)
ES	0.791 ± 0.33	1 ± 0.11	7.186 ± 1.2	57.08 ± 2.7	0.084	8.25 ± 1.2	11.9	

EMF	1.65 ± 0.94	2 ± 0.27	20.22 ± 1.5	198.4 ± 2.2	0.077	8.98 ± 1.1	12.98	

ESLN-3	4.21 ± 0.63	6 ± 0.17	79.2 ± 1.1	866.6 ± 3.5	0.0263	26.65 ± 0.78	38.48	391.69*

*Relative bioavailability with respect to EMF.

(i) AUMC: area under the first moment curve; MRT: mean residence time; *K*_el_: elimination rate constant; RB: relative bioavailability.

(ii) EMF, ES, and ESLN-3 are formulations containing Efavirenz equivalent to 10 mg.

All data expressed as mean ± S.D, *n* = 6 (*P* ≤ 0.05).
